# Soft Tissue Responses to Orthodontic Treatment: Impact of Premolar Extraction on Diverse Growth Patterns

**DOI:** 10.7759/cureus.58077

**Published:** 2024-04-11

**Authors:** Arathi Murugesan, Navaneethan Ramasamy, Sruthi Harikrishnan

**Affiliations:** 1 Department of Orthodontics and Dentofacial Orthopedics, Saveetha Institute of Medical and Technical Sciences, Chennai, IND

**Keywords:** research, quality of life, retraction, extraction, growth pattern, soft tissue changes

## Abstract

Introduction: Alteration in facial soft tissue plays an important role in the esthetics of an individual. The first thing a patient wants from orthodontic treatment is how well he/she looks. The degree of soft tissue changes brought about by the retraction of teeth can be influenced by factors such as extraction pattern, muscle function, age, gender, weight, etc.

Aim: The aim of the study was to compare post-orthodontic soft tissue changes among different facial growth patterns in orthodontic patients undergoing extraction of first premolar teeth.

Methodology: Pre-treatment and post-treatment lateral cephalograms of 45 orthodontic patients who underwent therapeutic extraction of the first premolars were included in the study. They were divided into three equal groups based on their facial growth pattern, that is, average, horizontal, and vertical. Eight soft tissue cephalometric measurements were done in all the lateral cephalograms. Paired Student t-tests and analysis of variance (ANOVA) were conducted to statistically analyze the results. The significance level was set as 0.05.

Results: The paired Student t-test showed a *P*-value > 0.05 for lower anterior facial height in all three groups and for facial angle in vertical growers alone. ANOVA comparing the mean soft tissue changes among the three groups resulted in a *P*-value > 0.05 for all the measured parameters.

Conclusions: Facial growth patterns do not influence the extent of soft tissue profile changes in orthodontic patients treated with extraction of first premolars.

## Introduction

Approximately 75% of individuals seek orthodontic treatment, to improve their facial symmetry and aesthetic appearance [1]. A pleasing facial profile and esthetics are known to have a positive influence on people's interpersonal relationships and self-esteem [2,3]. Lips play a major role in facial esthetics as protruded incompetent lips are seen as unpleasant by patients [4]. Lip positions can be altered by orthodontic treatment. Thus, providing functional occlusion along with improved facial esthetics becomes the prime goal of orthodontic treatment.

Orthopedic and functional treatment, orthodontic camouflage, and orthognathic surgery are the various treatment options available to improve the skeletal and soft tissue relationships of orthodontic patients. Orthopedic and functional therapy is used to treat growing patients, while orthognathic surgery is done in adult patients where camouflage cannot produce an efficient treatment result [5]. Patients with relatively minor skeletal irregularities may benefit from orthodontic treatment [6]. Considering camouflage in bimaxillary protrusion cases, the need to extract the teeth or not remains a debatable issue.

Particularly intriguing are the changes in facial soft tissues that occur after bicuspid teeth are removed and the anterior teeth are retracted. A multitude of researchers have evaluated the impact of orthodontic therapy involving tooth extraction on facial aesthetics [7-11]. However, their findings remain contentious. Premolar extraction has been linked to a flatter profile by some studies, but others have found no such link after a thorough clinical and radiological evaluation [9,12-14]. A few studies have been conducted to investigate the role of other factors in facial soft tissue alterations in orthodontic patients undergoing extraction. Therefore, this study aimed to compare the post-orthodontic soft tissue changes among different vertical facial growth patterns in orthodontic patients undergoing extraction of first premolars.

## Materials and methods

In our retrospective study, we meticulously selected participants based on well-defined inclusion and exclusion criteria to ensure homogeneity in the sample and the relevance of the findings. The study encompassed a demographic of patients aged 18 to 30 years, reflecting the typical orthodontic adult patient population. Eligibility was strictly limited to those who underwent orthodontic treatment involving the therapeutic extraction of both upper and lower first premolars, ensuring consistency in treatment type across our sample. Further qualifying criteria necessitated completed treatment and an anchorage loss of less than 25%, thereby selecting successful orthodontic outcomes that did not require additional corrective procedures. Conversely, certain conditions and scenarios warranted exclusion from the study to isolate the effects of orthodontic intervention on soft tissue changes. Patients presenting with craniofacial anomalies were excluded to avoid confounding anatomical variables. Additionally, individuals who had undergone alternative space-gaining methods, other than the extraction of all first premolars, were not considered to prevent confounding effects from varying treatments. Those whose orthodontic treatment resulted in an anchorage loss exceeding 25% were also omitted, as were individuals with supernumerary or missing teeth, which could inherently affect dental and facial structures. Finally, the exclusion criteria extended to patients with a history of previous orthodontic treatment, facial surgery, or trauma, to eliminate preexisting conditions that might skew the intended focus on changes directly attributable to the first premolar extraction process.

The determination of sample size was a crucial component of our study design, influencing both the power of our statistical tests and the validity of our conclusions. We calculated the required sample size using G*Power software, as this program is widely recognized for its comprehensive approaches to power analysis for a variety of statistical tests. Our calculations were based on an estimated effect size. We thought that our main outcome measure would have a moderate effect size, which was in line with previous research in this area, and suggested that the study would find a meaningful clinical difference. The effect size was specified as Cohen's d, which was selected due to its applicability to comparing two means under the assumption of normally distributed data. Based on an extensive literature review and preliminary data, we anticipated a moderate effect size of *d* = 0.5. This expectation is in line with Cohen's benchmarks, where 0.2 denotes a small effect, 0.5 represents a medium effect, and 0.8 defines a large effect. For our sample size estimation, the following parameters were set: an α level (type I error rate) of 0.05, ensuring a 5% chance of falsely detecting an effect; and a power (1 - β) of 0.80, allowing a 20% probability of type II error, i.e., failing to detect an actual effect. With an effect size of 0.5, power analysis indicated that a total of 45 subjects would be required to adequately test our hypothesis [14]. The study sample consisted of 45 randomly selected individuals' pre- and posttreatment lateral cephalograms. Based on the vertical face growth pattern, the samples were classified into three groups. Patients in Group 1 had an average growth pattern, patients in Group 2 had a horizontal growth pattern, and patients in Group 3 had a vertical growth pattern.

The growth pattern was determined using Tweed’s Frankfort Mandibular Plane Angle (FMA) and Jarabak’s ratio [15]. The range of FMA for average growth pattern was taken as 220 to 280. An angle less than 220 indicated a horizontal growth pattern and an angle greater than 280 indicated a vertical growth pattern. Jarabak’s ratio of 62 to 65 indicated average growth, more than 65 indicated short face, and less than 62 indicated long face. The participants whose FMA and Jarabak's ratios disagreed were excluded from the research.

All patients were treated with a pre-adjusted edgewise appliance with a 0.022 x 0.028-inch slot and MBT bracket prescription. The wire sequencing for leveling and aligning was the same. En-mass anterior retraction was done using friction mechanics on a 0.019 x 0.025-inch SS archwire. Cephalograms were taken before and after treatment, with the patient's head in its natural head position. All the cephalometric tracings and measurements were done by the same investigator using FACAD cephalometric software. The soft tissue parameters assessed are listed in Table 1. Intra-examiner error was assessed by repeating the measurements on 10 radiographs, which were randomly selected after two weeks, and assessing the intraclass correlation coefficient (ICC). An ICC value of 0.8 to 0.9 was obtained, which indicated an excellent level of agreement.

**Table 1:** Various soft tissue cephalometric measurements used in this study.

Measurements	Definitions
Ls-SL (mm)	Distance between the most prominent point on the upper lip (labrale superioris; Ls) to the S line (SL), the line drawn from soft tissue pogonion (Pg’) to the middle of the *S* formed by the lower border of the nose
Li-SL (mm)	Distance between the most prominent point on the lower lip (labrale inferioris; Li) to SL
Li-H line (mm)	Distance between Li to H line, the line formed by the union of Pg’ and Ls
Lower anterior facial height (mm)	Distance from subnasale (Sn) to soft tissue menton (Me’)
Facial angle (degree)	The angle between the Frankfort horizontal plane and the soft tissue facial plane (soft tissue nasion to Pog’)
H-line angle (degree)	The angle between the soft tissue facial plane and the H line
Nasolabial angle (degree)	The angle between the tangent to the nasal base and upper lip through Sn
Upper lip angle (degree)	The angle between the tangent to the upper lip and a true vertical line passing through Sn

Statistical analysis

IBM SPSS Statistics for Windows, Version 20.0 (IBM Corp., Armonk, NY) was used for all statistical analysis. We calculated the average and standard deviation using descriptive statistics. The normality of the data was assessed using the Shapiro-Wilk test. Wilcoxon or paired Student t-tests were used to compare statistically significant post-treatment improvements between the two groups. One-way analysis of variance (ANOVA) was used to compare the mean values of the three groups before treatment and their mean differences before and after treatment. For all statistical tests, the level of significance was fixed at 0.05.

## Results

Table 2 shows the demographics of our samples by age and gender. We used ANOVA to examine the severity of the pretreatment soft tissue condition across the three groups (Table 3). Out of all the eight measurements, only lower anterior facial height (LAFH) showed a significant difference.

**Table 2:** Descriptive statistics for subjects. Average growth pattern: 22° to 28° FMA. Horizontal growth pattern: <22° FMA. Vertical growth pattern: >28° FMA. FMA, Frankfort Mandibular Plane Angle

Groups	Age (Mean +/- SD) years	Male/Female
Group 1 (Average growth pattern)	22.8 +/- 4.49	7.00/8.00
Group 2 (Horizontal growth pattern)	21.9 +/- 4.83	7.00/8.00
Group 3 (Vertical growth pattern)	21.2 +/- 2.66	8.00/7.00

**Table 3:** Descriptive statistics and comparison of pretreatment values of the three groups (ANOVA). *P*-value < 0.05 indicates a statistically significant difference. SD, standard deviation; ANOVA, analysis of variance

Measurements	Group 1 average growth pattern		Group 2 horizontal growth pattern		Group 3 vertical growth pattern		Significance (*P*) value
	Mean	SD	Mean	SD	Mean	SD	
Ls-SL (mm)	1.21	1.57	0.31	1.14	1.02	1.79	0.249
Li-SL (mm)	4.95	2.12	3.33	2.92	3.66	2.96	0.235
Li-H line (mm)	4.63	1.99	2.83	1.69	3.12	2.45	0.057
Lower anterior facial height (mm)	63.89	5.57	62.54	4.93	84.99	27.14	0.001*
Facial angle (°)	88.83	3.59	90.71	2.30	89.33	3.38	0.060
H-line angle (°)	19.15	2.99	18.71	2.56	19.11	2.54	0.886
Nasolabial angle (°)	96.91	13.88	93.14	11.35	95.87	12.72	0.704
Upper lip angle (°)	23.89	9.16	23.37	8.11	21.25	7.74	0.659

**Figure 1:**
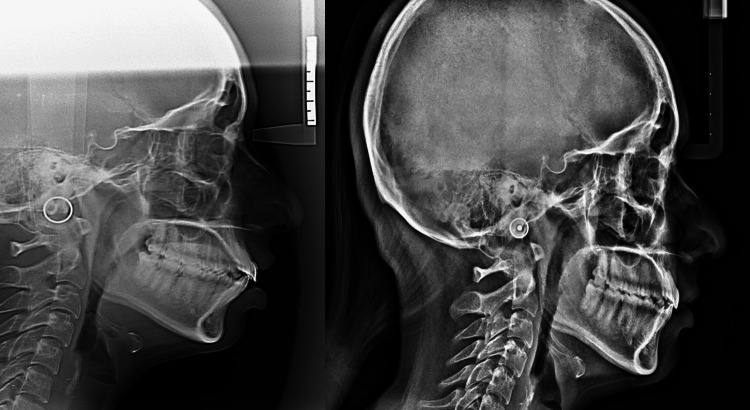
Pre- and post-lateral cephalograms in group 1.

**Figure 2:**
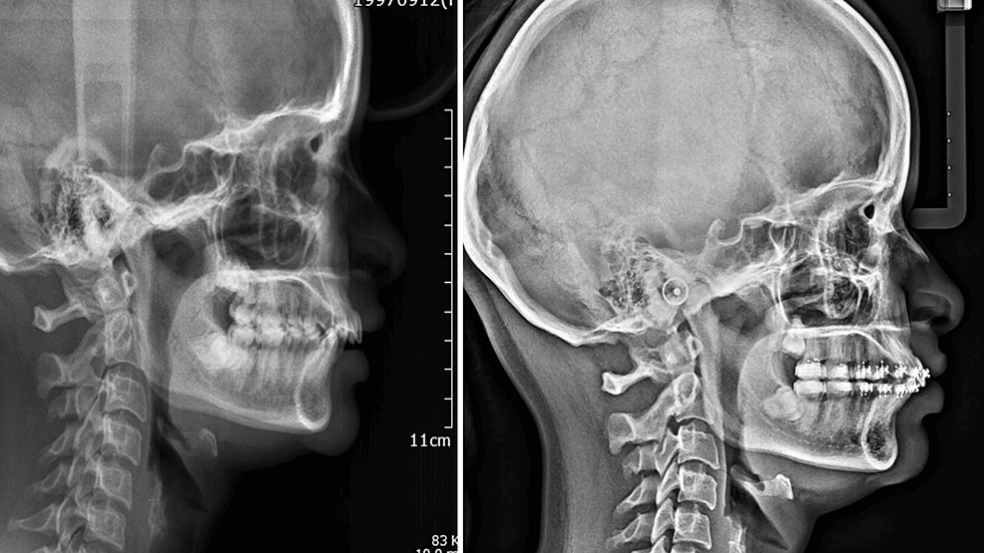
Pre- and post-lateral cephalograms in group 2.

**Figure 3:**
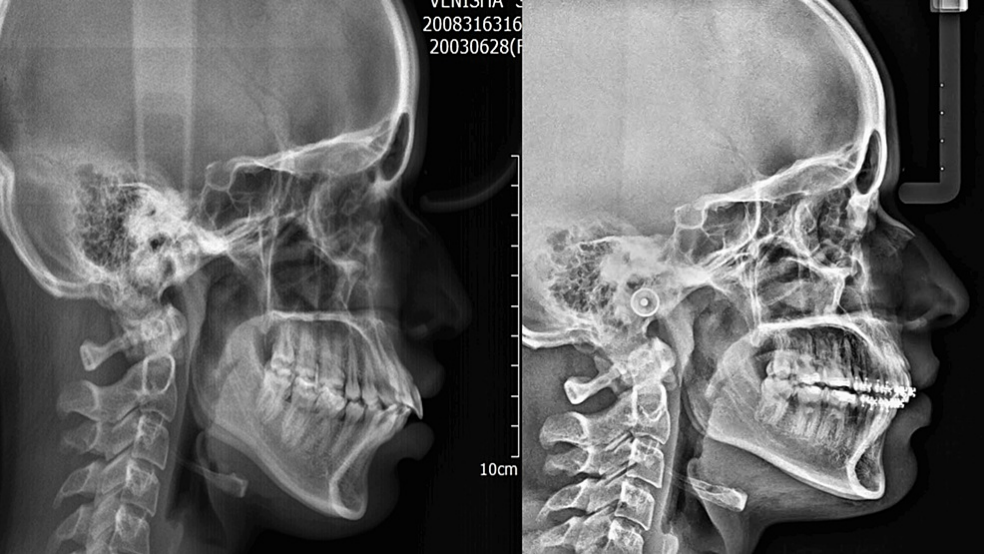
Pre- and post-lateral cephalograms in group 3.

A normal distribution was found in the data using the Shapiro-Wilk test. Tables 4, 5, and 6 show the average, horizontal, and vertical growth patterns together with their corresponding means, standard deviations, and significance values (paired t-test) for both the pre- and posttreatment periods. A decrease in posttreatment values of Ls-SL, Li-SL, Li-H line, facial angle, H line angle, and upper lip angle and an increase in LAFH and nasolabial angle was noticed in all three groups. However, there were no statistically significant changes (*P*-value > 0.05) in the posttreatment LAFH measurements in the three groups and facial angle measurements in group 3. Pre- and post-cephalograms for group 1 are shown in Figure 1. Pre- and post-cephalograms for group 2 are shown in Figure 2. Pre and post-cephalograms for group 3 are shown in Figure 3.

**Table 4:** Mean, standard deviation, and paired t-test significance for pre- and posttreatment values for group 1 (average growth pattern). SD= Standard deviation; p value < 0.05 indicates statistically significant difference

Measurements	Pretreatment values		Posttreatment values		Changes (Pretreatment - Posttreatment values)		Significance (*P*) value
	Mean	SD	Mean	SD	Mean	SD	
Ls-SL (mm)	1.21	1.57	-1.21	1.4	2.42	0.99	0.001*
Li-SL (mm)	4.95	2.12	2.49	1.87	2.45	1.73	0.001*
Li-H line (mm)	4.63	1.99	2.72	1.28	1.91	1.97	0.002*
Lower anterior facial height (mm)	63.89	5.57	65.43	5.14	-1.55	3.41	0.101
Facial angle (°)	88.83	3.59	87.33	3.83	1.49	2	0.012
H-line angle (°)	19.15	2.99	15.97	2.60	3.18	1.88	0.001*
Nasolabial angle (°)	96.91	13.88	110.43	18.64	-13.51	12.56	0.001*
Upper lip angle (°)	23.89	9.16	11.98	13.44	11.91	8.38	0.001*

**Table 5:** Mean, standard deviation, and paired t-test significance for pre- and posttreatment values for group 2 (horizontal growth pattern). *P*-value < 0.05 indicates a statistically significant difference. SD, standard deviation

Measurements	Pretreatment values		Posttreatment values		Changes (Pretreatment - Posttreatment values)		Significance (*P*) value
	Mean	SD	Mean	SD	Mean	SD	
Ls-SL (mm)	0.31	1.14	-1.41	1.24	1.73	1.12	0.001*
Li-SL (mm)	3.33	2.92	1.48	3.00	1.85	1	0.001*
Li-H line (mm)	2.83	1.69	1.51	2.14	1.32	1.17	0.001*
Lower anterior facial height (mm)	62.54	4.93	64.29	6.5	-1.75	3.99	0.111
Facial angle (°)	90.71	2.30	90.22	3.23	0.49	2.70	0.045
H-line angle (°)	18.71	2.56	14.17	2.21	4.53	1.55	0.001*
Nasolabial angle (°)	93.14	11.35	108.54	13.28	-15.4	10.36	0.001*
Upper lip angle (°)	23.37	8.11	13.17	9.78	10.19	4.07	0.001*

**Table 6:** Mean, standard deviation, and paired t-test significance for pre- and posttreatment values for group 3 (vertical growth pattern). *P*-value < 0.05 indicates a statistically significant difference. SD, standard deviation

Measurements	Pretreatment values		Posttreatment values		Changes (Pretreatment - Posttreatment values)		Significance (*P*) value
	Mean	SD	Mean	SD	Mean	SD	
Ls-SL (mm)	1.02	1.79	-1.41	1.55	2.43	1.47	0.001*
Li-SL (mm)	3.66	2.96	1.11	2.45	2.55	1.36	0.001*
Li -H line (mm)	3.12	2.45	1.39	1.81	1.73	1.96	0.004*
Lower anterior facial height (mm)	84.99	27.14	87.61	28.32	-2.62	5.13	0.068
Facial angle (°)	89.33	3.38	89.19	3.47	0.15	1.50	0.710
H-line angle (°)	19.11	2.54	15.33	2.20	3.78	1.45	0.001
Nasolabial angle (°)	95.87	12.72	110.47	14.76	-14.60	11.12	0.001*
Upper lip angle (°)	21.25	7.74	12.98	10.30	8.27	6.85	0.001*

Results of ANOVA comparing the soft tissue changes among the three groups showed that there was no significant difference in the soft tissue changes between the groups (Table 7). Quantitative changes produced in the soft tissue parameters were similar in all the groups.

**Table 7:** Comparison of differences in soft tissue changes among the three groups (ANOVA). *P*-value > 0.05 indicates no statistically significant difference. ANOVA, analysis of variance

Measurements	Group 1 average growth pattern		Group 2 horizontal growth pattern		Group 3 vertical growth pattern		Significance (*P*) value
	Mean	SD	Mean	SD	Mean	SD	
Ls-SL (mm)	2.42	0.99	1.73	1.12	2.43	1.47	0.201
Li-SL (mm)	2.45	1.73	1.85	1	2.55	1.36	0.335
Li-H line (mm)	1.91	1.97	1.32	1.17	1.73	1.96	0.641
Lower anterior facial height (mm)	-1.55	3.41	-1.75	3.99	-2.62	5.13	0.764
Facial angle (°)	1.49	2	0.49	2.70	0.15	1.50	0.083
H-line angle (°)	3.18	1.88	4.53	1.55	3.78	1.45	0.088
Nasolabial angle (°)	-13.51	12.56	-15.4	10.36	-14.60	11.12	0.902
Upper lip angle (°)	11.91	8.38	10.19	4.07	8.27	6.85	0.338

## Discussion

One of the most crucial requirements for successful orthodontic therapy is the modification of soft tissues. An increase in facial muscle function is associated with a higher horizontal craniofacial growth pattern than a vertical growth pattern [16-18]. However, there is little evidence assessing the influence of craniofacial growth patterns on soft tissue changes after orthodontic treatment. This study aimed to determine the differences in soft tissue changes following the extraction of all four first premolars and subsequent space closure in average, horizontal, and vertical facial growth patterns.

Historically, it was thought that non-extraction treatments led to more desirable facial changes. This perception could stem from incorrect diagnoses, overtreatment, or a lack of orthognathic surgery understanding [19]. Yet, studies in the 21st century by Kim et al., Rathod et al., Zierhut et al., and Freitas et al. indicated no significant difference in posttreatment facial profiles between individuals treated with or without extractions [14,16,17,20,21]. Further research focusing on asymmetric extraction patterns reveals that symmetric extractions tend to produce greater profile changes compared to asymmetric ones.

In this study, the pre-treatment soft tissue characteristics were matched across the three groups, except for the difference in the LAFH. Matching was conducted to minimize confounding variables and potential bias. All groups showed significant soft tissue changes posttreatment. Despite a decrease in various posttreatment measurements and an increase in LAFH and nasolabial angle, these changes were found to be aesthetically acceptable. However, increased LAFH in vertical growers could be perceived as less desirable, necessitating careful management to minimize the extrusion of molars during space closure.

The study reports that facial growth patterns do not influence soft tissue changes following orthodontic treatment with extraction. However, there are two significant limitations of this study: its retrospective design and the duration, which may not capture the complete spectrum of changes over time. Cephalometric measurements used for soft tissue analysis might affect the reliability of reported changes.

Despite these limitations, the study offers valuable insights into short-term soft tissue changes posttreatment. For future research, a prospective design with a longer duration could provide a more comprehensive understanding of changes over time. Advanced imaging and 3D analysis could also enhance the accuracy and reliability of soft tissue profile changes in future studies.

## Conclusions

These findings sharply contradict the notion that facial growth patterns significantly influence the degree of soft tissue profile changes in orthodontic patients who have undergone extraction of their first premolars. The collected and analyzed data indicate remarkable uniformity in the soft tissue alterations, regardless of the diverse growth patterns observed among the subjects.

In conclusion, this study's results make a valuable contribution to the existing knowledge in orthodontics by offering a nuanced perspective on the relationship between facial growth patterns and soft tissue profile changes following the extraction of first premolars. As the field progresses, these insights will likely inspire further research and contribute to the ongoing refinement of evidence-based orthodontic practices.
